# Supplementary material for: Rational programming of history-dependent logic in cellular populations
Source: Nat Commun. 2020 Sep 21;11:4758. doi: 10.1038/s41467-020-18455-z (PMC7506022; doi:10.1038/s41467-020-18455-z)
Supplement: Supplementary file 1 — Supplementary Information [file 41467_2020_18455_MOESM1_ESM.docx]

**Supplementary Materials for**

**Rational programming of history-dependent logic in cellular populations.**

**Authors:**

Ana Zúñiga†^1^, Sarah Guiziou†^1#^, Pauline Mayonove^1^, Zachary Ben Meriem^2^, Miguel Camacho^1^, Violaine Moreau^1^, Luca Ciandrini^1, 3^, Pascal Hersen^2,4^, and Jerome Bonnet^1*^

**Affiliations:**

^1^ Centre de Biochimie Structurale (CBS). INSERM U154, CNRS UMR5048, University of Montpellier, France.

^2^Laboratoire Matière et Systèmes Complexes, UMR 7057 CNRS & Université Paris Diderot, 10 rue Alice Domon et Léonie Duquet, 75013, Paris, France

^3^ Laboratoire Charles Coulomb (L2C), University of Montpellier & CNRS, France.

^4^ Laboratoire Physico Chimie Curie, UMR168, Institut Curie, Paris, France.

^#^ Current address: Department of Biology, University of Washington, Seattle, Washington 98195, USA

† These authors contributed equally to this work

* to whom correspondence should be addressed: jerome.bonnet@inserm.fr

**This PDF file includes:**

Supplementary Text

Figs. S1 to S21

Table S1

**Supplementary Text**

**Supplementary Text S1. Minimization of history-dependent circuits using Boolean logic devices**

The number of strains required for implementing history-dependent gene-expression programs can be reduced using Boolean logic devices. Indeed, gene-expression programs independent of the history of occurrence of inputs are implementable using Boolean logic [^41^](https://paperpile.com/c/nxeBIs/BT2Z5). Some history-dependent gene-expression programs are decomposable into Boolean logic function(s) and history-dependent subprogram(s). The combination of history-dependent and Boolean logic devices allows a reduction in the number of strains required for the implementation of some history-dependent gene-expression programs. We created an algorithm in Python to automate this simplification. We generated all Boolean functions and converted each truth table into a lineage tree. To implement history-dependent programs, we tested if any Boolean functions can be extracted from this program. If the use of Boolean devices leads to an implementation with an equal or reduced number of strains, the design is saved. We then obtained as output a list of designs based on Boolean and/or history-dependent devices implementing the input program with the minimal number of strains possible. We applied this brute-force method to all 3-input/1-output programs, totaling 65,536 programs. This strategy allows for a reduction in the number of strains for the implementation of 20% of these programs. The results did not significantly reduce the median number of strains required for the implementation of history-dependent programs, however 48% of 3-input/1-output programs are decomposable using Boolean programs while minimizing the number of strains (Fig. S15).

**Figure S1**


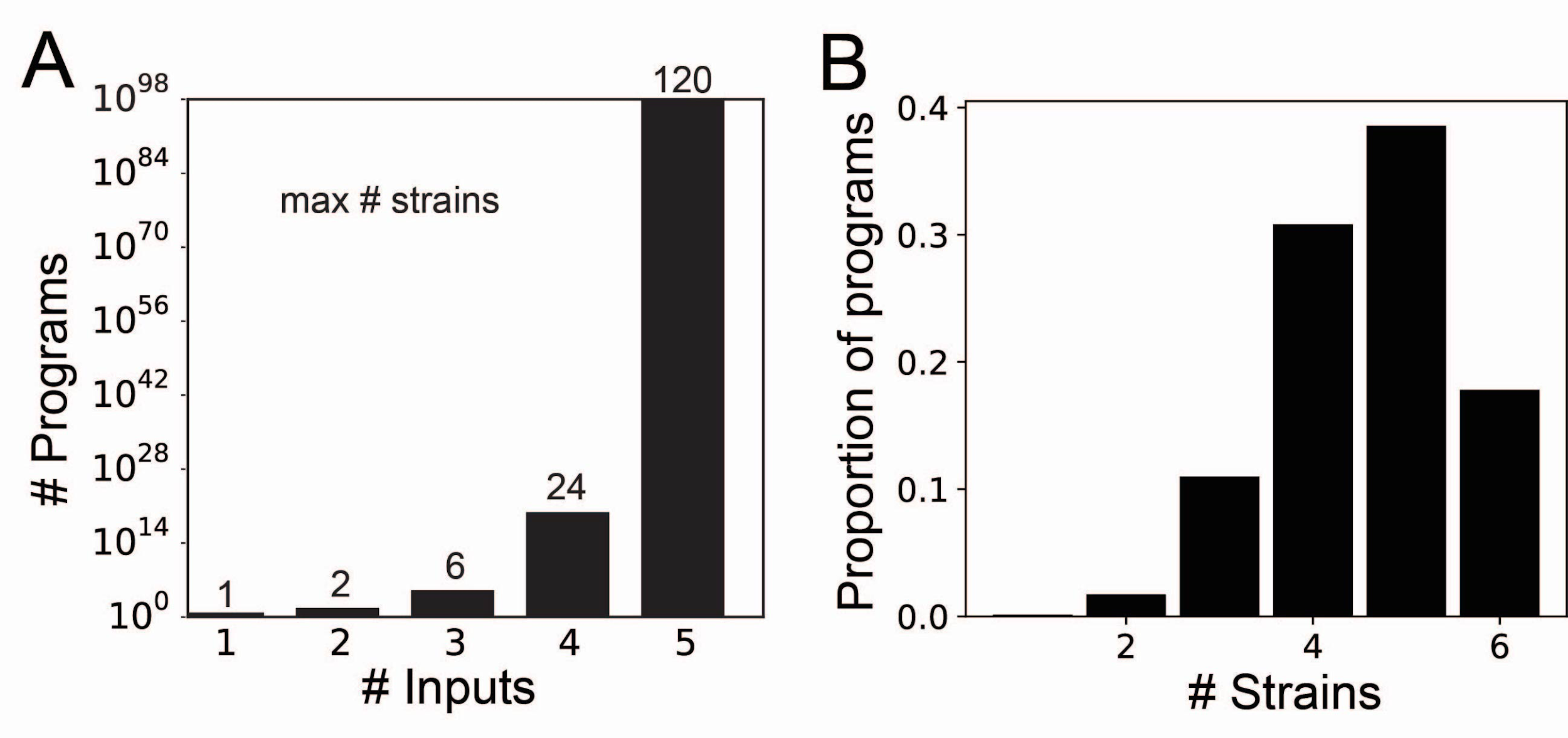


**Figure S1. Number of strains needed to implement a history-dependent program.** (A) Number of single-output history-dependent programs and maximum number of strains needed for 1 to 5 inputs. The bar graph represents the number of single-output history-dependent programs from 1 to 5 inputs and the number at the top of each bar corresponds to the maximum number of strains required for the implementation of these programs. See materials and methods for detailed equations. (B) Distribution of the number of strains required for the implementation of 3-input history-dependent programs. Y-axis represents the number of programs and X-axis the number of strains required for its implementation. The distribution of strains corresponds to all 3-input 1-output history-dependent programs. Data was obtained using a Python algorithm which generates the designs of all 3-input 1-output programs.

**Figure S2**


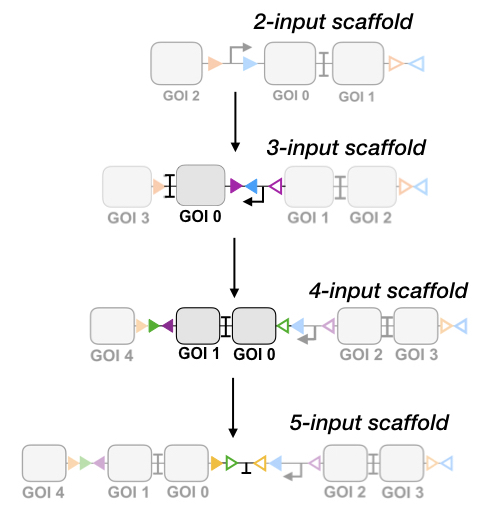


**Figure S2: Iterative design of scaffolds for up to 5 inputs.** The design of scaffold for 3-, 4- and 5 inputs are based on the N-1 input scaffold (N being the number of input). This iterative design is highlighted in this figure as the part of the scaffold different from the N-1 scaffold is represented in bold color. For each incrementation, a new integrase site pair corresponding to the first input of the lineage (purple for 3 inputs, green for 4 inputs and yellow for 5 inputs) is added in inversion orientation. Between this integrase site pair, one integrase site corresponding to the second input of the lineage is placed and inverted in comparison to the N-1 design to be in excision orientation. Additional GOI positions and terminators are also added.

**Figure S3**

**
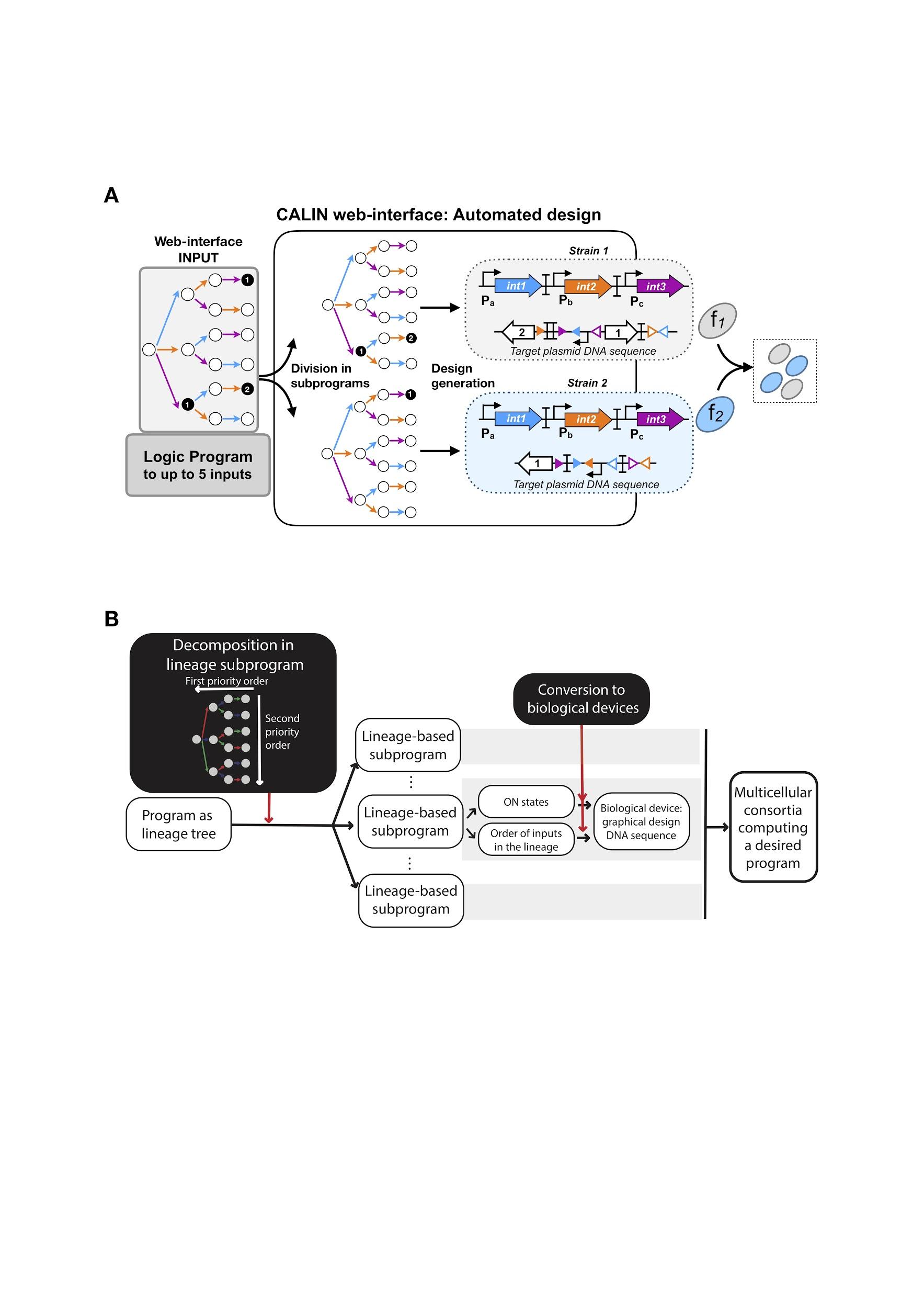
**

**Figure S3. Automated design of history dependent programs using CALIN.** Automated design of history-dependent programs using a web-interface **(A)** and diagram software of CALIN **(B)** The Python program takes as input a history-dependent program written as a lineage tree. This program is decomposed into sub-programs, and the decomposition is performed by preferentially extracting subprograms with ON state at the extremity of the tree (corresponding to state with the highest number of inputs present). For each subprogram, the algorithm identifies the identity of ON states and the order of the inputs in the lineage. Based on this information, the biological design is obtained and the graphical design for integrase and history-dependent DNA devices with its corresponding DNA sequences are given. The full program composition is implemented by combining the population of strains with different subprograms.

**Figure S4**

**
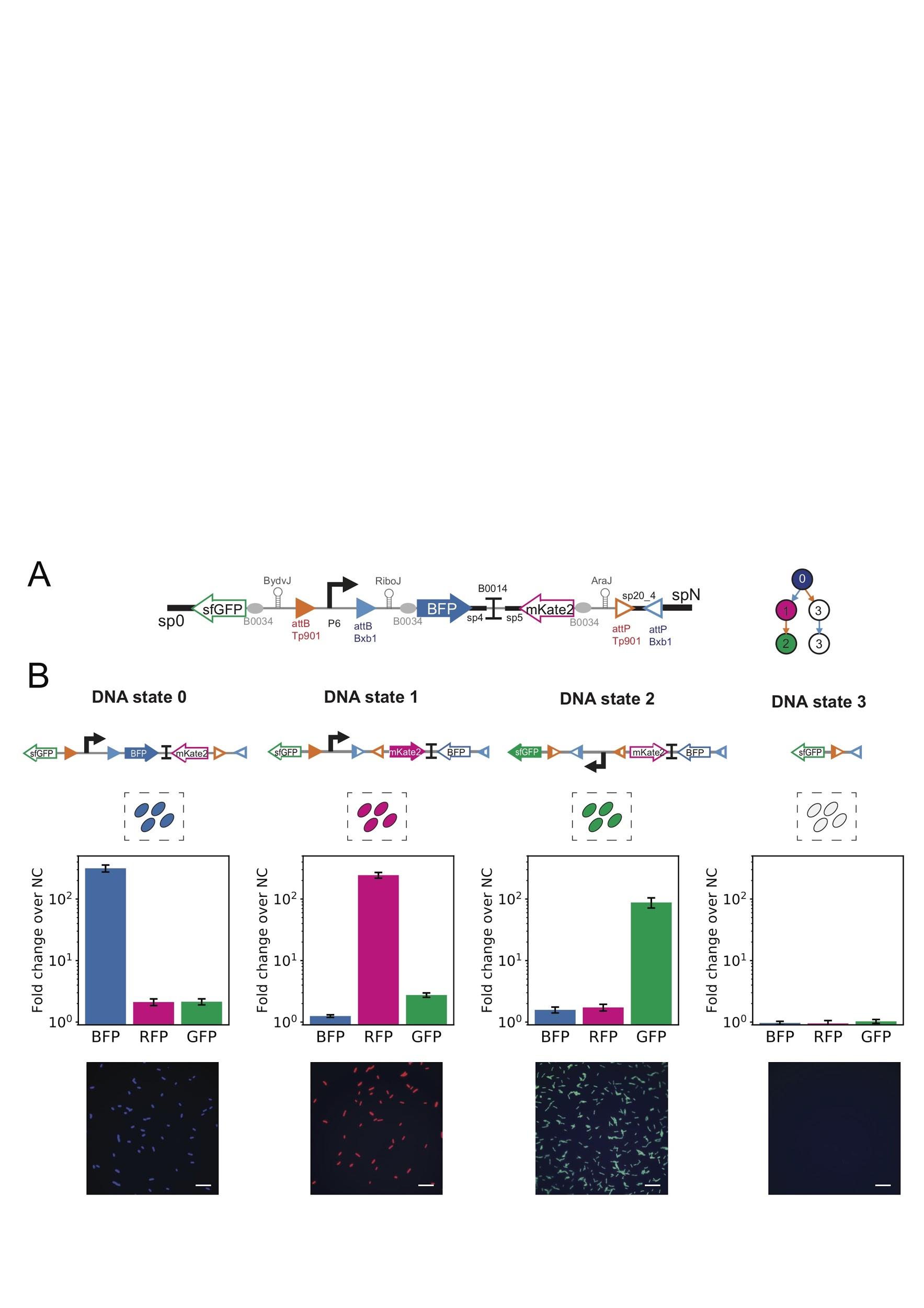
Figure S4. Design and characterization of 2-input scaffold by OSIRiS. (A)** Detailed design of the 2-input scaffold for the lineage A then B with Bxb1 for input A and Tp901 for input B. As output gene, we used BFP, RFP and GFP and in 5’UTR of each gene we placed a ribozyme and B0034 RBS to have a translation isolated from genetic context. We used P6 as promoter and B0014 as bidirectional terminator between BFP and RFP coding sequences. We added 40bp spacers for gibson assembly (sp0, sp4, sp5 and spN) and a 20bp spacer between two juxtaposed integrase sites (sp20_4). **(B)** Characterization of the 2-input scaffold DNA states using OSIRiS by flow-cytometry. We characterized each recombination state in a flow-cytometry by measurement of GFP, RFP and BFP fluorescence intensities. The bar graph corresponds to the fold change over the negative control (strain without fluorescent protein) for each fluorescence channel. The error bar corresponds to the standard deviation between the fold change obtained from 2 biological replicates. The microscopy images correspond to merge images of the GFP, RFP and BFP channels. Bars, 20 μm.

**Figure S5**

**
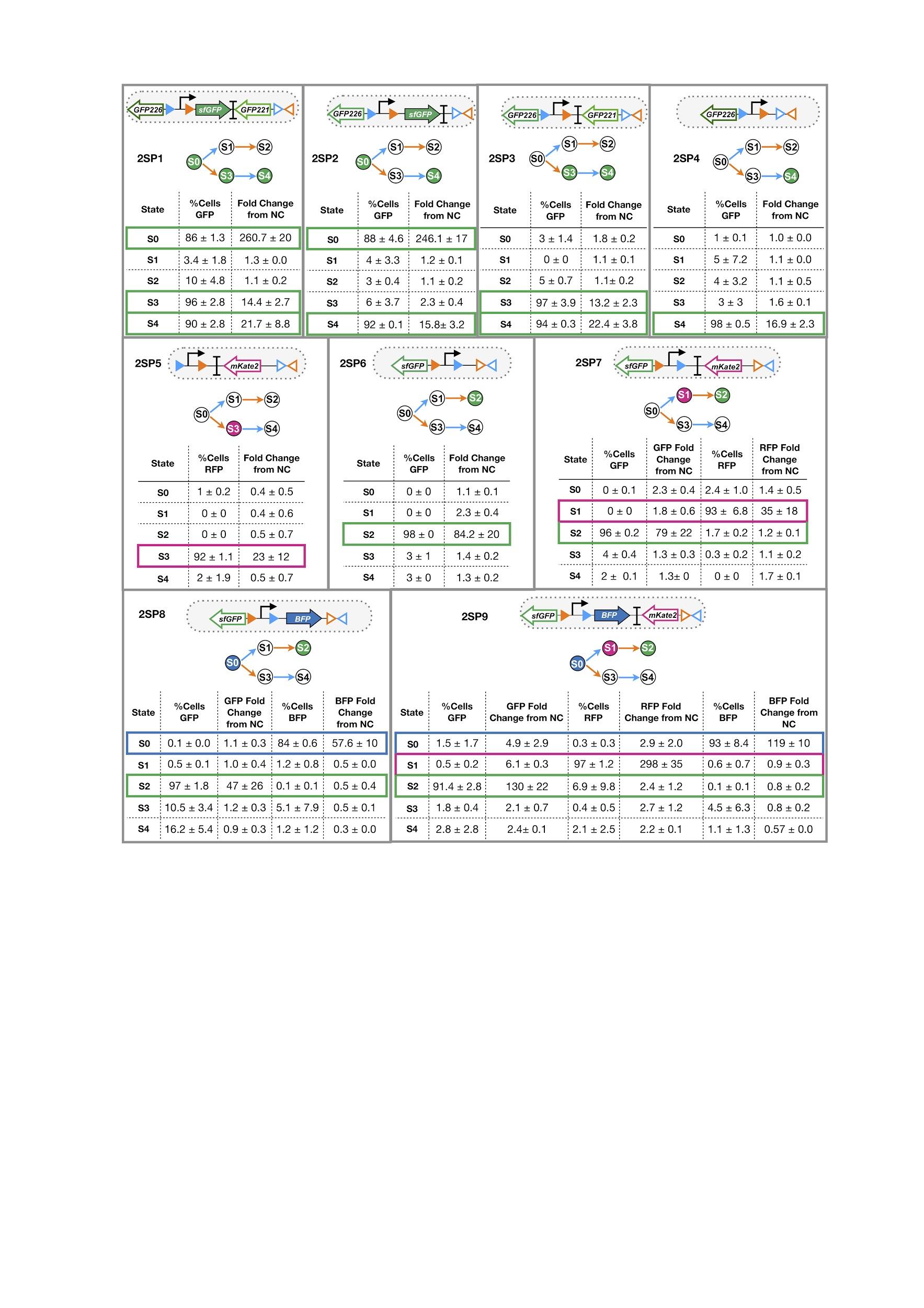
**

**Figure S5. Percentage of cells and fluorescence fold change in each different input state for 2-input single-lineage programs.** The lineage tree for each program and its corresponding genetic diagram are represented. Each table shows the percentage of cell and fold change in fluorescence intensity over the negative control (strain without fluorescent protein) measured after sequential induction series with different order-of-occurrences of inputs. Values correspond to averages and standard deviations for three different experiments performed in triplicate on three different days.

**Figure S6**

**
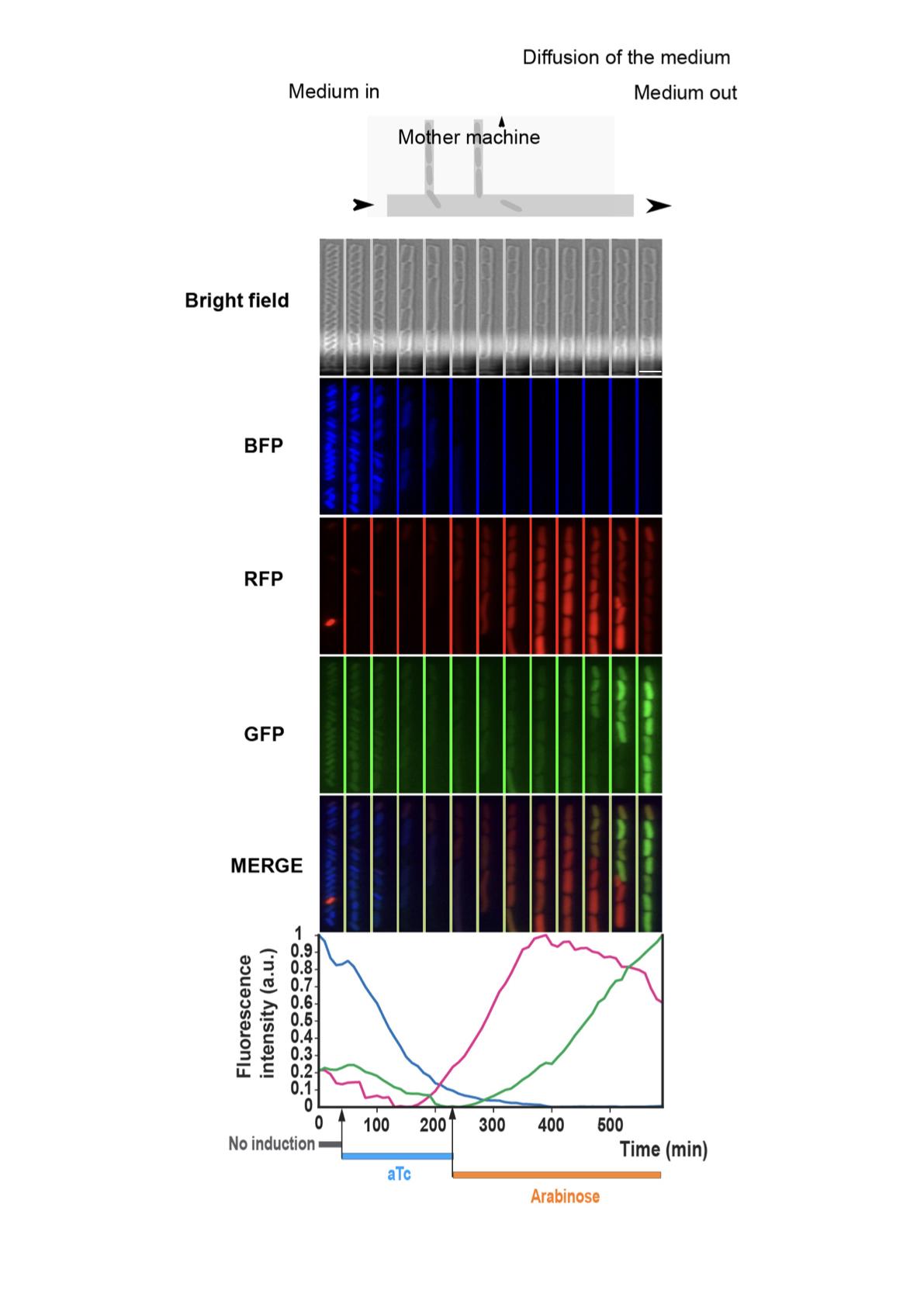
**

**Figure S6. Switching kinetics of 2SP9 single-lineage program.** Cells were grown in a mother machine microfluidic for 10 h and analyzed by time-lapse microscopy. Cells were grown for 30 min without induction, followed by 3 h of aTc induction. Next, the medium was changed and arabinose induction was performed. Microscopy images show the fluorescence expression of cells in the mother machine during this time. The graph shows the kinetic curves of the mean of fluorescence intensity normalized over the maximum fluorescence intensity for each channel (GFP, RFP and BFP). Ten channels were analyzed from one experiment. Bar, 2 μm.

**Figure S7**


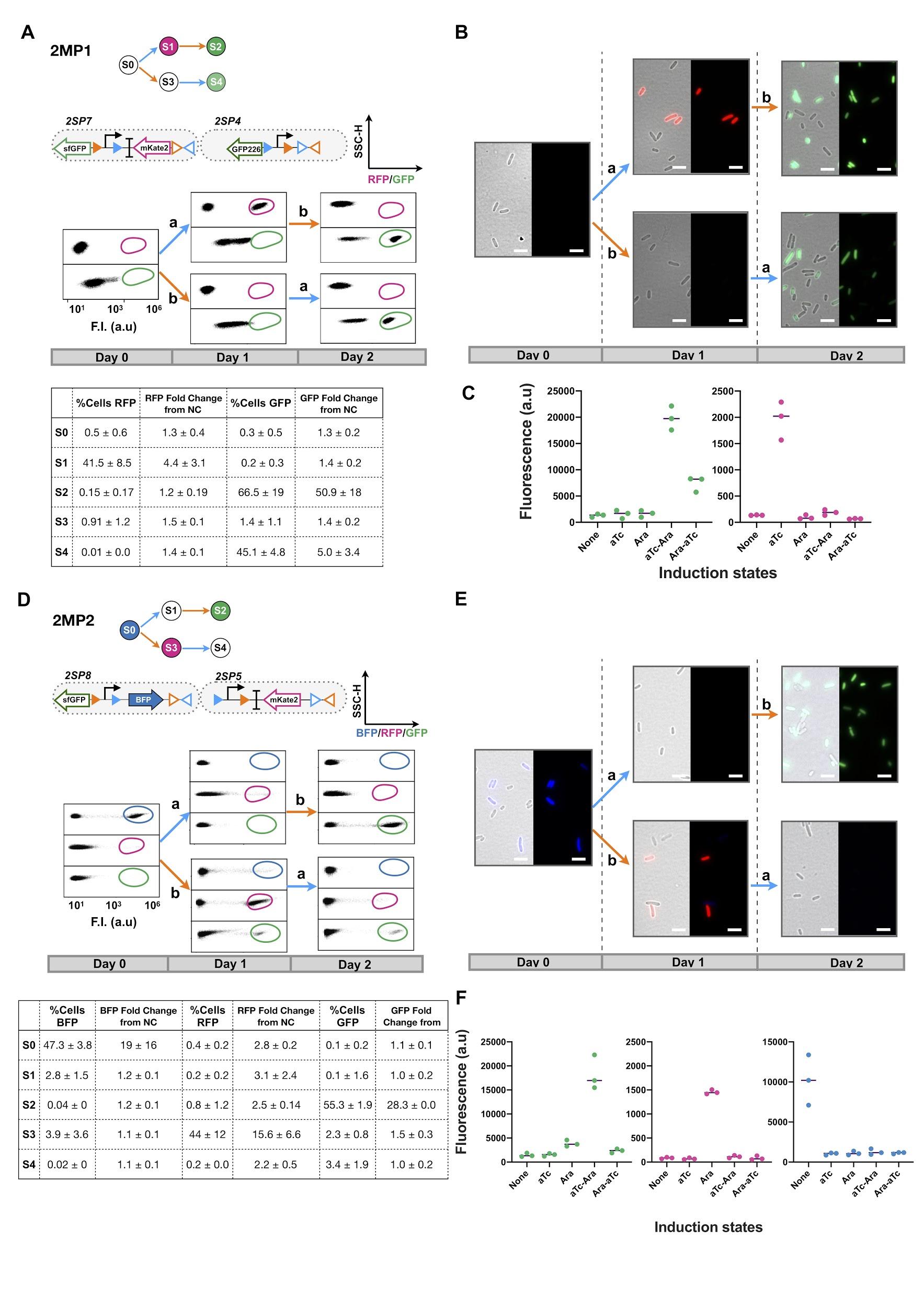


**Figure S7. Cell population analysis from lineages of 2-input multicellular programs.** Flow cytometer (A and D), microscopy analysis (B and E), and dot plots from plate reader experiment (C and F) of populations in 2-input multicellular programs 2MP1 and 2MP2, respectively. Statistical data of fluorescence fold change and cell percentage on fluorescence channel, for each subpopulation in different input states are shown. Scatter plots showing SSC-H (side scatter height) versus fluorescence intensity (F.I), are representative of three independent experiments channels for BFP, RFP and GFP, from top to bottom are plotted. Positive controls expressing fluorescence reporter genes were used to set the gates for each fluorescence channel. The microscopy images correspond to the merged images of the GFP, RFP, and BFP channels with bright-field (left) and without (right). Bars, 10 μm. Dot plots correspond to data distribution of the bar chart in Figure 3d-e, from plate reader measurements for 2-input multicellular programs. Dots correspond to the mean value of fluorescence intensity in arbitrary units (a.u) for each fluorescent channel (green, GFP; red, RFP and; blue, BFP), with different and linear scales each.

**Figure S8**

**
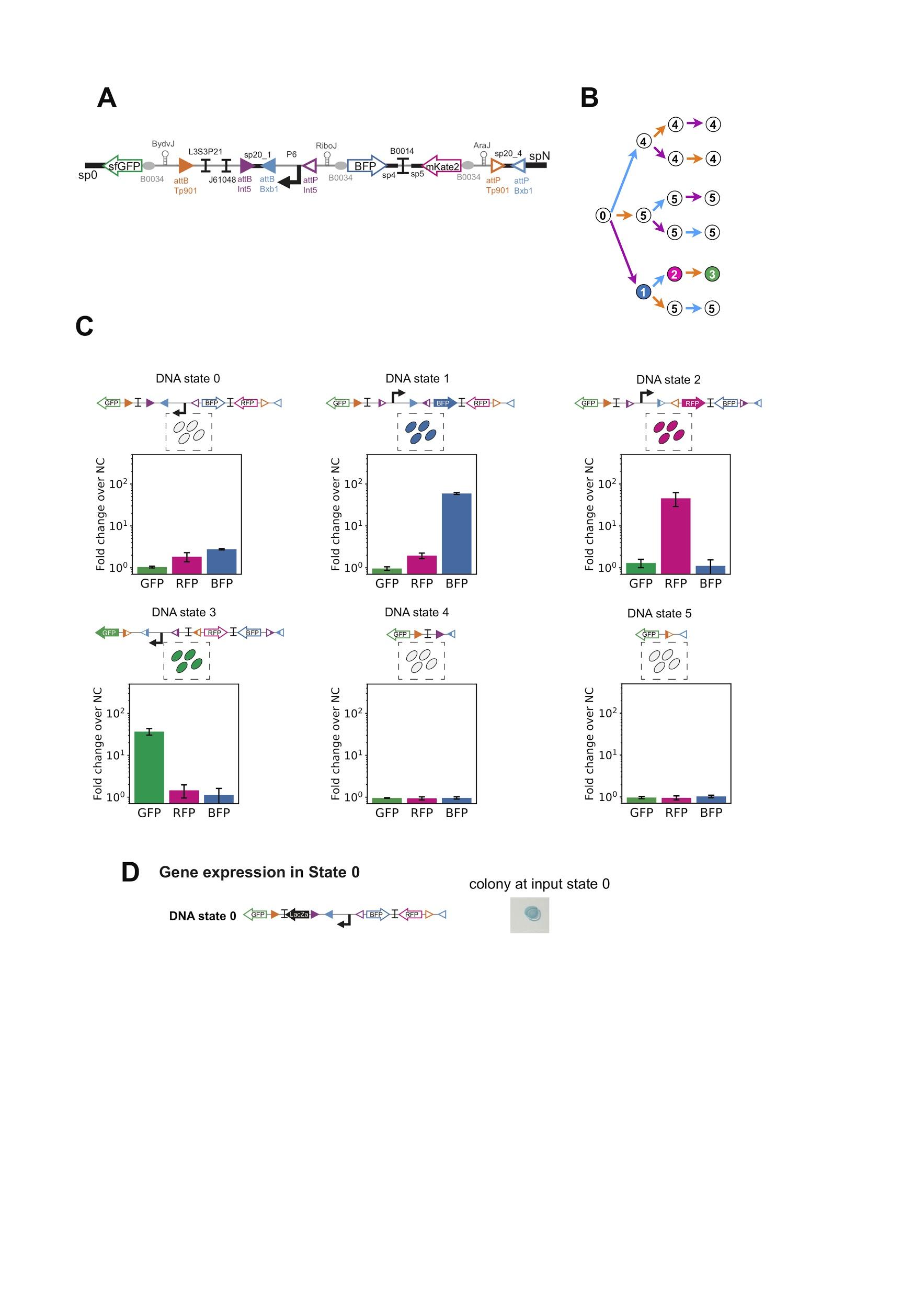
**

**Figure S8. Final design and characterization of 3-input scaffold by OSIRiS. (A)** Detailed design for the final 3-input scaffold. In this design, no gene is expressed in the initial input state. **(B)** 3-input lineage tree corresponding to the final 3-input scaffold. The color of each node corresponds to the expected phenotype and the number in the node to the corresponding DNA state of each input state. **(C)** Characterization of the final 3-input scaffold and its recombination intermediates DNA states by flow-cytometry. We characterized each DNA state by measurement of GFP, RFP and BFP fluorescence intensities. The bar graph corresponds to the mean value of fold change over the negative control (strain without fluorescent protein) for each channel from three experiments with three replicates per experiment. The error bars correspond to the standard deviation from fold changes obtained in three separate experiments. **(D)** Additional designed device with *lacZ alpha* gene expression at DNA state 0, in the 3-input scaffold. The media was supplemented with X-gal, to obtain blue colonies.

**Figure S9**

**
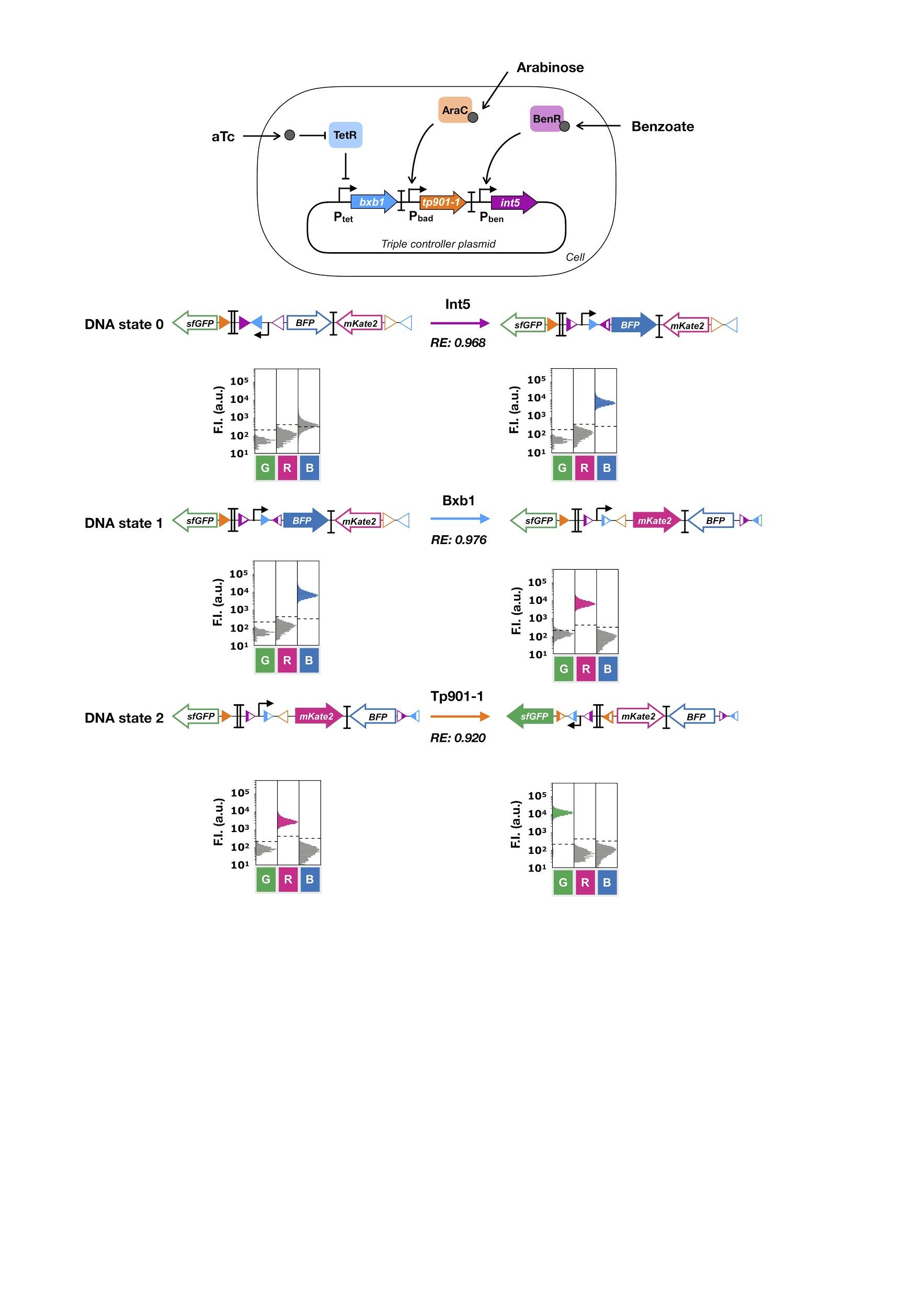
**

**Figure S9. Functional characterization of the triple recombinase controller.** Cells harboring the triple controller plasmid were transformed with scaffolds encoding the DNA states for 3-input scaffold generated by OSIRiS, to test the recombination efficiency and the resulting state transitions controlled by Integrase 5, Bxb1 and Tp901-1. Cells were induced with either benzoate 100 µM for Int5, aTc 200 ng/uL for Bxb1 and Arabinose 0.7% for Tp901-1. *RE*, is the recombination efficiency obtained from the % of cells expressing the fluorescent reporter gene after induction of recombinase under the experimental conditions described in methods. For characterization every co-transformed strain was induced for 16 hours. Each histogram shows fluorescent reporter genes expressed as a result of different induction conditions. Each histogram is representative of two different experiments measured by flow cytometry.

**Figure S10**

**
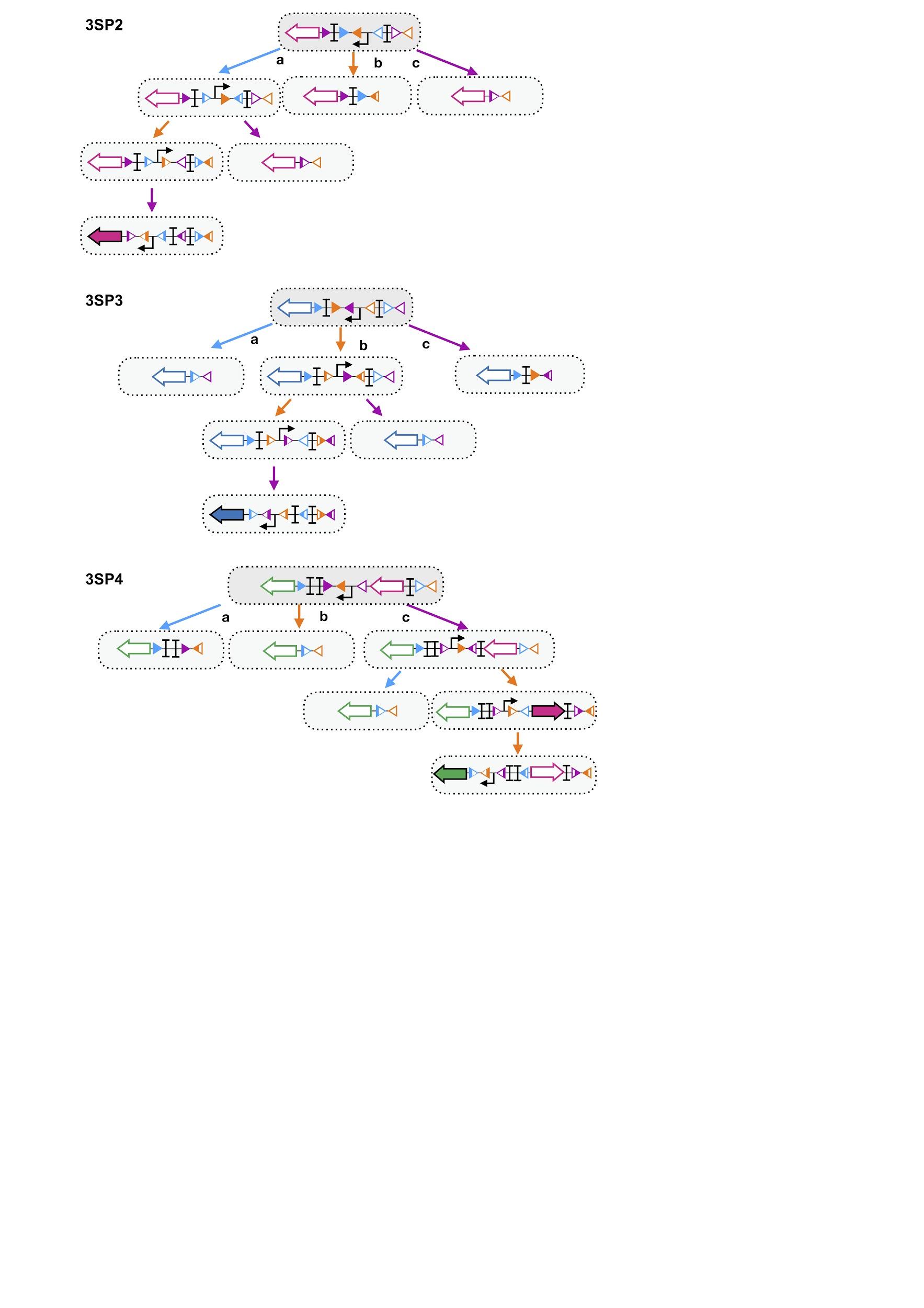
**

**Figure S10. DNA states diagrams of 3-input single-lineage programs used in this work.**  DNA and gene expression states of 3SP2 3SP3 and 3SP4 programs. Each program has six different DNA states.

**Figure S11**

**
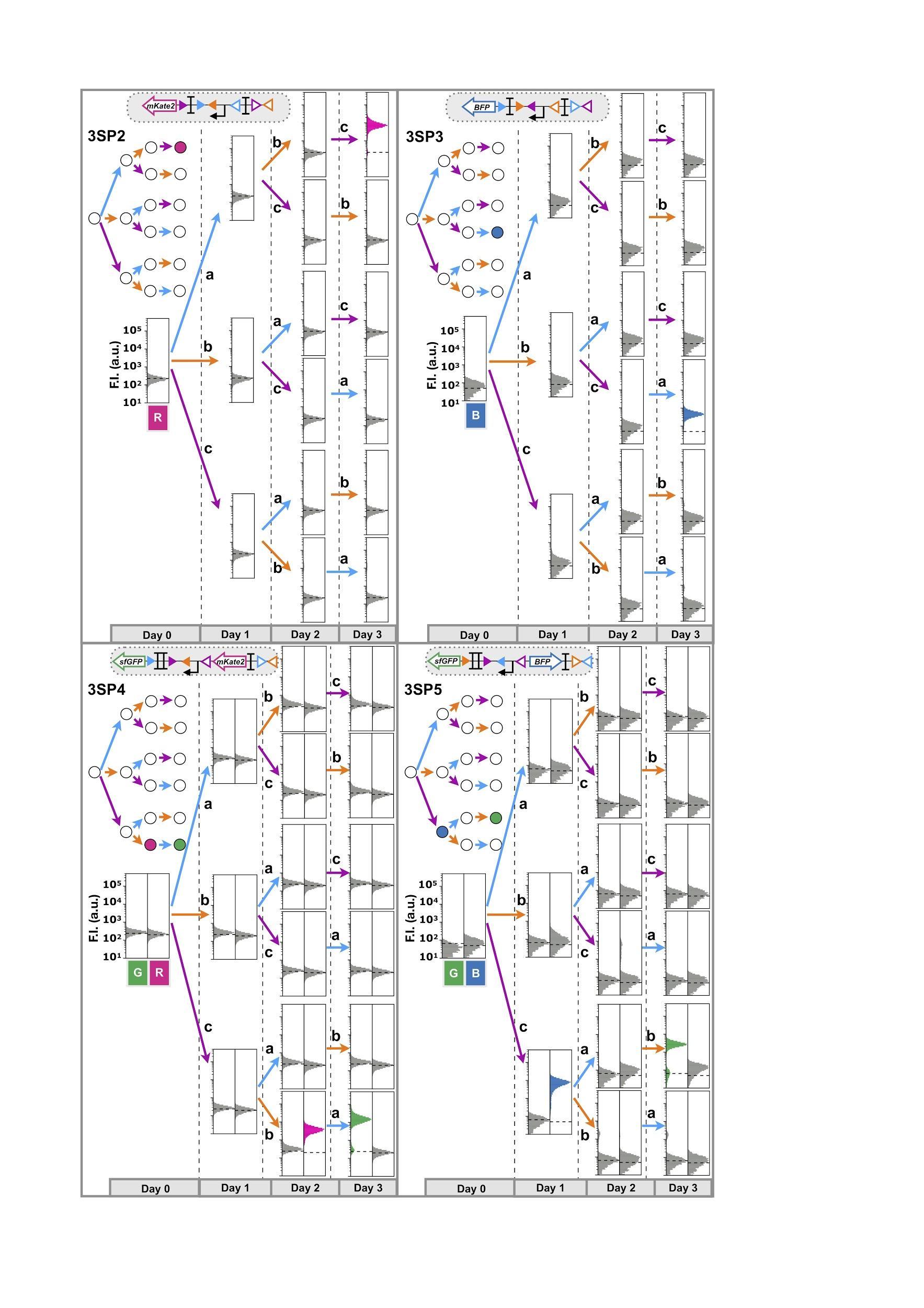
**

**Figure S11. Histograms of 3-input single-lineage programs characterization flow cytometry.** Four history-dependent single-lineage programs were implemented and characterized. We co-transformed each 3-input program with the triple controller plasmid. Bxb1 expression is induced by aTc (input a), Tp901 by arabinose (input b) and Int5 by benzoate (input c). The lineage tree for each program and its corresponding genetic device are represented. For characterizing the system, cells were sequentially induced three times for 16 hours each, with different order-of-occurrences of inputs. Each histogram shows fluorescent reporters expressed in different states. All experiments were performed in triplicate three times on three different days. A representative example is depicted here

**Figure S12**

**
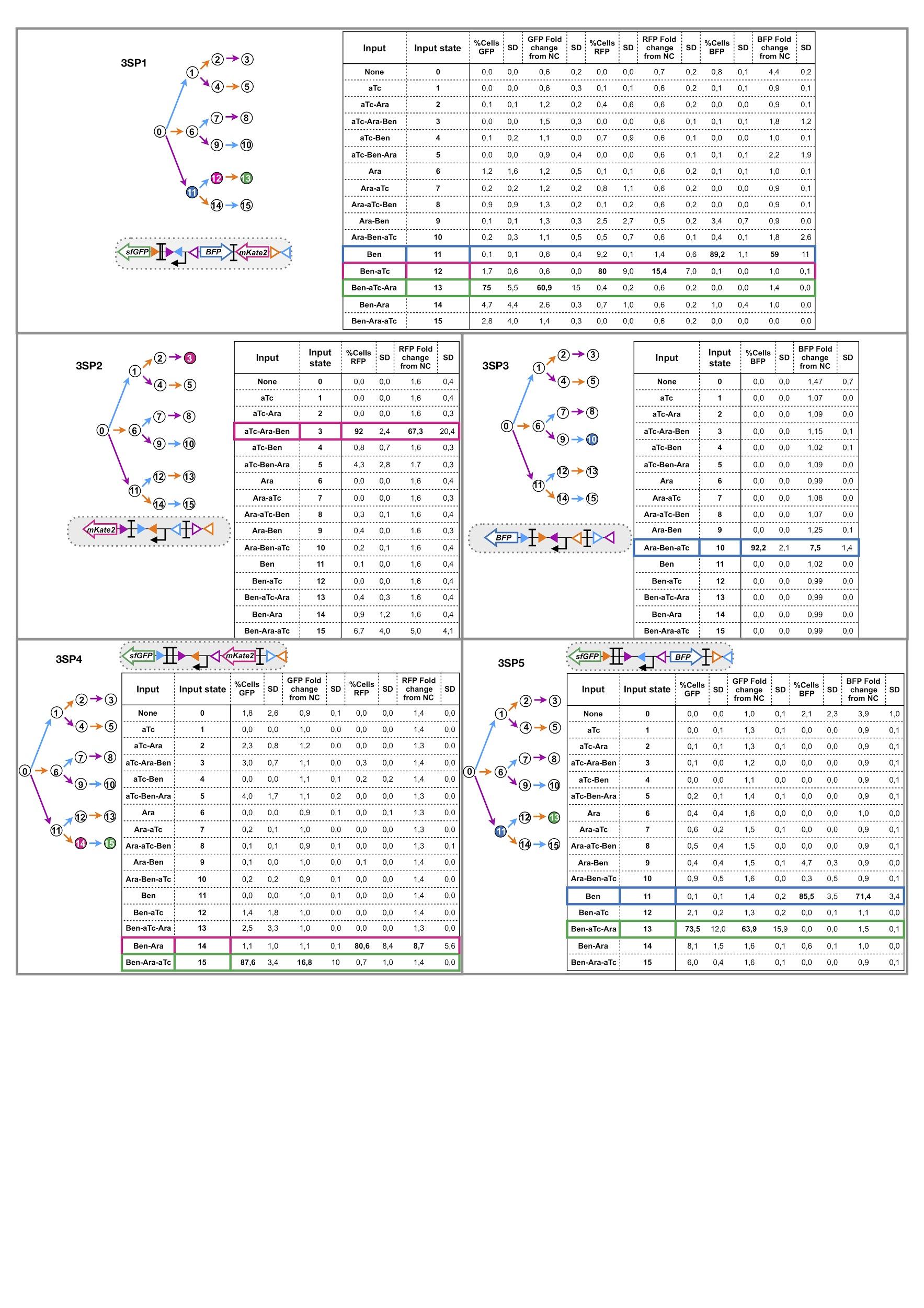
**

**Figure S12. Fluorescence fold change and percentage of cells on different lineage input-states for 3-input single-lineage programs.** The lineage tree for each program and its corresponding genetic device are represented. Each table shows the percentage of cells and fold change of the media fluorescence intensity over the negative control (strain without fluorescent protein) as a result of the sequential induction with different order-of-occurrences of inputs. Values correspond to averages and standard deviations for three different experiments performed in triplicate on three different days.

**Figure S13**

**
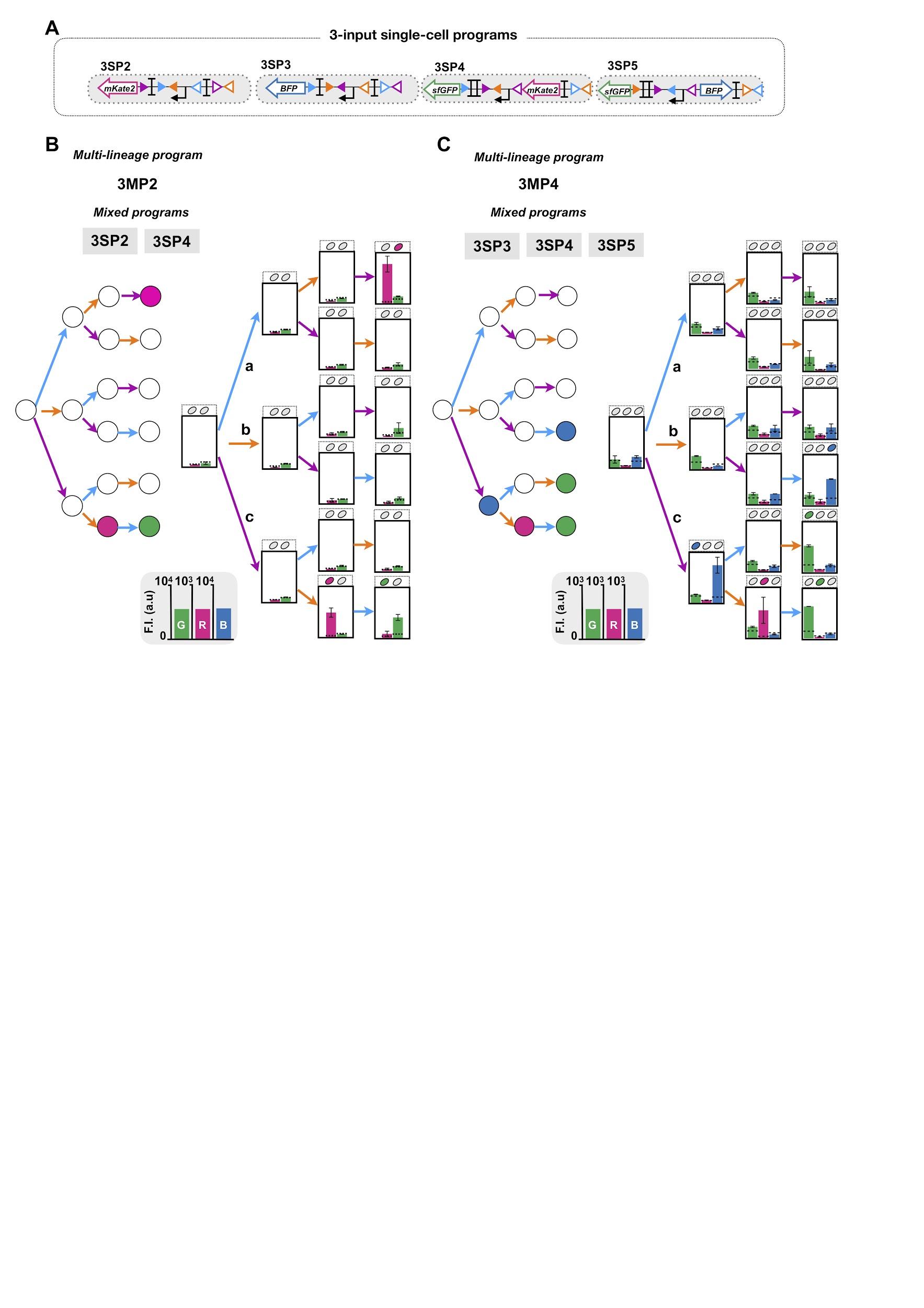
**

**Figure S13. Characterization of 3-input multicellular programs.** Design and characterization of 3-input single-lineage programs operating in various lineages (**A**). Multicellular programs (**B**-**C**) were composed by mixing two or three single-lineage programs, **B** and **C**, respectively. The inputs are represented by letters, a (blue) for aTc (expression of Bxb1 Integrase), b (orange) for arabinose (expression of integrase TP901-1) and c (purple) for benzoate (expression of Int5). Strains were mixed in equal proportions and grown during 16 hours followed by sequential inductions with different order-of-occurrences of inputs. The bar graph corresponds to the mean value of fluorescence intensity (F.I) in arbitrary units (a.u) for each fluorescent channel (GFP, RFP and BFP), with different and linear scales each. The error bars correspond to the standard deviation for three different experiments measured by plate reader. The dotted line indicates the autofluorescence of negative control strain.

**Figure S14**

**
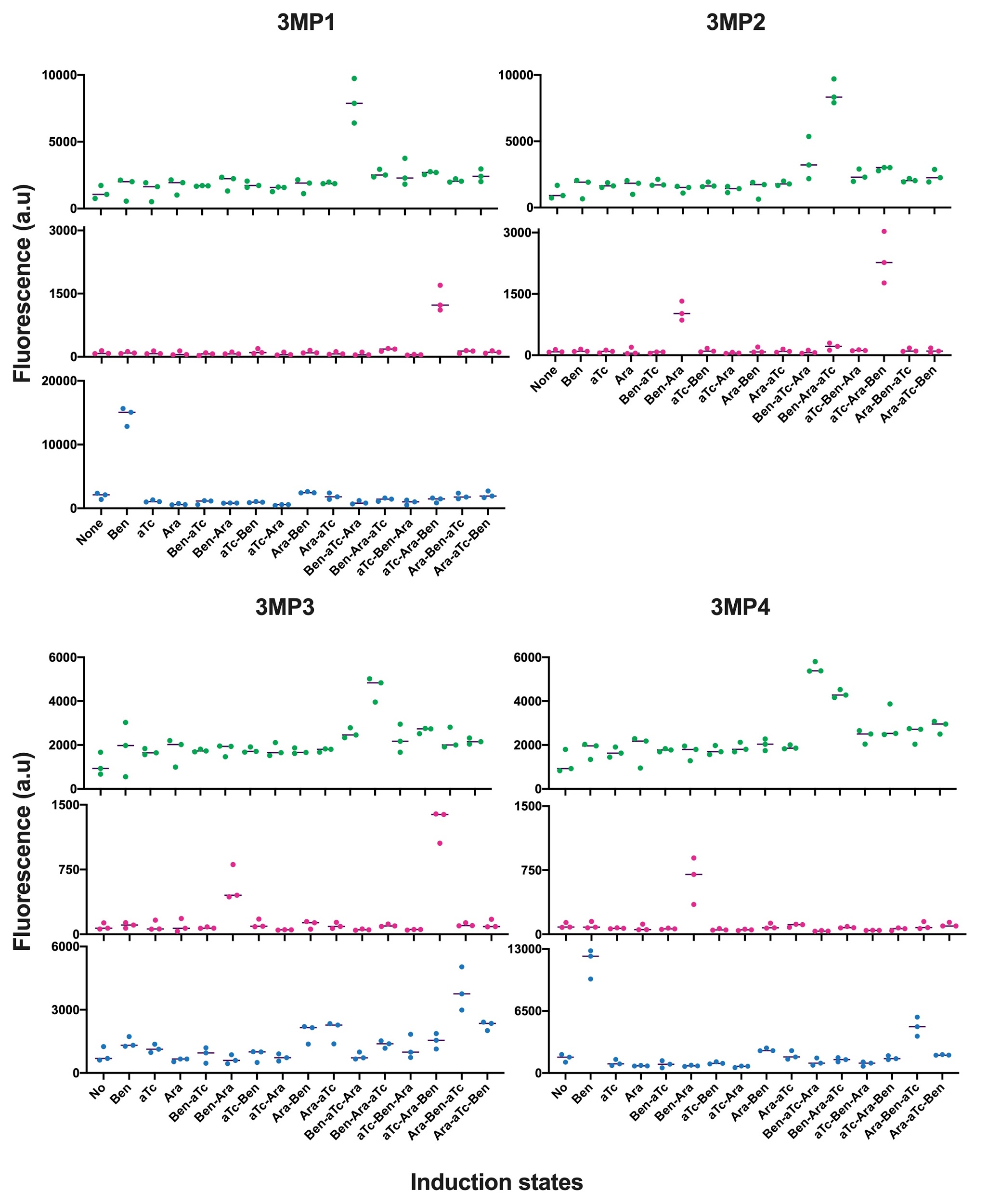
**

**Figure S14. Characterization of 3-input multicellular programs.** Dot plots correspond to the data distribution of bar chart in Figure 6 and S13, from plate reader measurements, for 3-input multicellular programs. Dots correspond to the mean value of fluorescence intensity in arbitrary units (a.u), from three different experiments, for each fluorescent channel (green, GFP; red, RFP and; blue, BFP), with different and linear scales each.

**Figure S15**

**
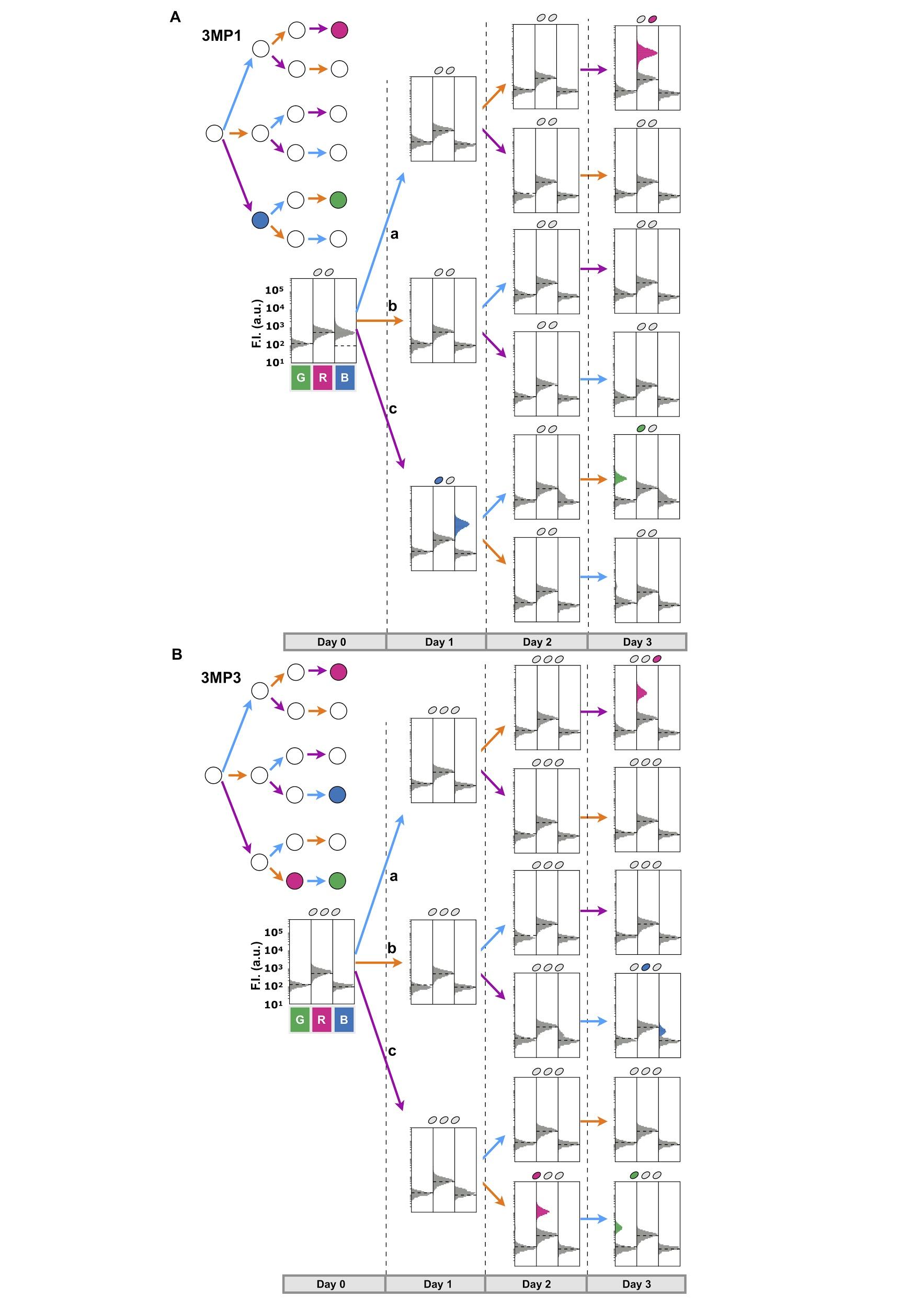
**

**Figure S15. Flow cytometry characterization of 3-input multicellular programs.** The flow cytometry analysis allows us to observe the percentage of population ON and OFF for each state. Both multicellular programs 3MP1 and 3MP3 were implemented using different single-lineage strains. The lineage trees for each program is represented. The inputs are represented by letters, *a* for aTc inducing Bxb1 Integrase and *b* for arabinose inducing Tp901-1 integrase. To implement each program the strains were mixed in similar proportions, grown for 16 hours and sequentially induced with different order-of-occurrences of inputs. Each histogram shows the expression of fluorescent reporters expressed at different induction states. A representative example is depicted here.

**Figure S16**

**
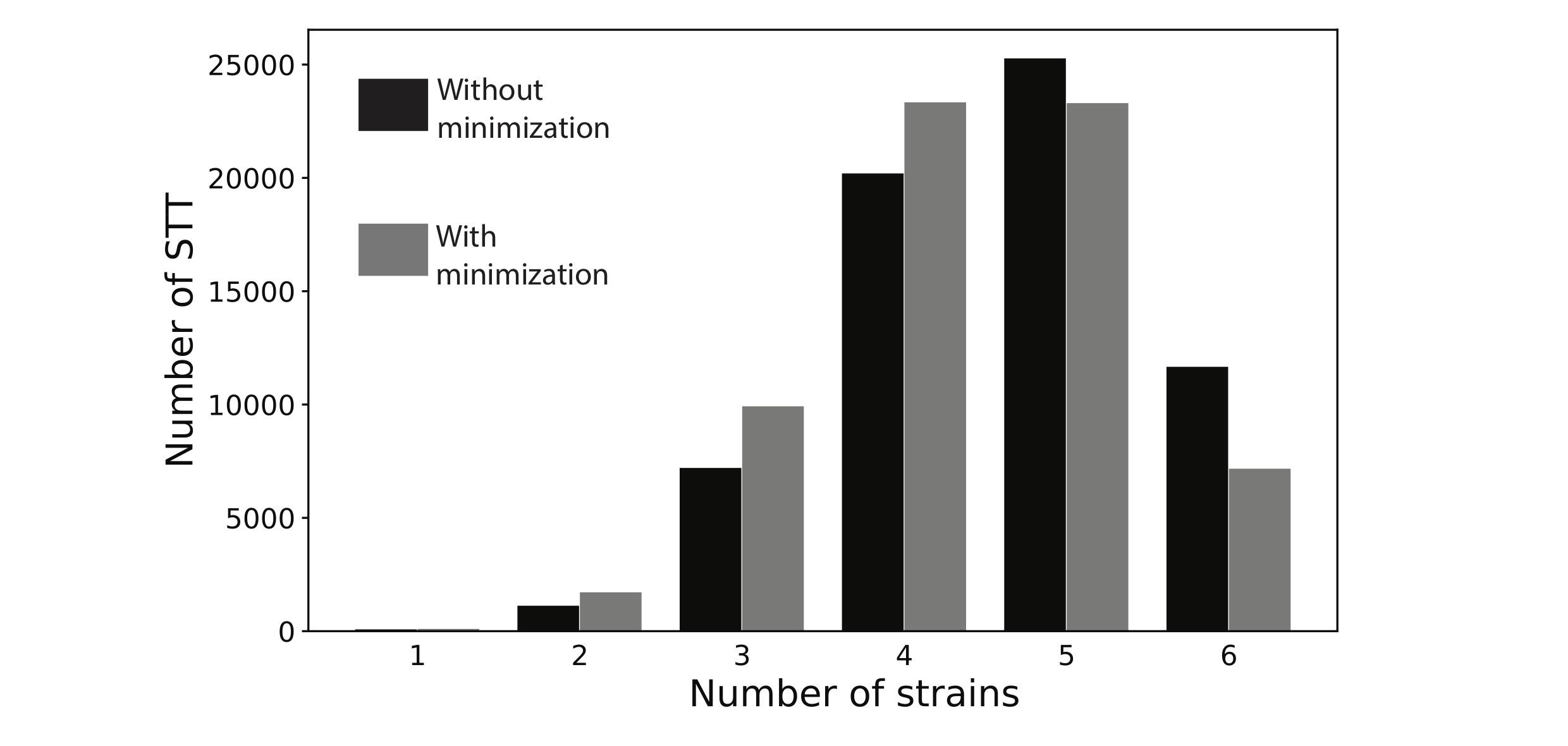
**

**Figure S16. Distribution of the number of strains required for the implementation of 3-input history-dependent programs.** Y-axis represents the number of sequential truth table (STT) and X-axis the number of strains required for its implementation. The distribution of strains corresponds to all 3-input 1-output history-dependent programs. Bars show the data without minimization (black bars), and with minimization (grey bars) using Boolean logic devices. Data were obtained using a Python algorithm which generates the designs with the two different strategies for all programs.

**Figure S17**

**
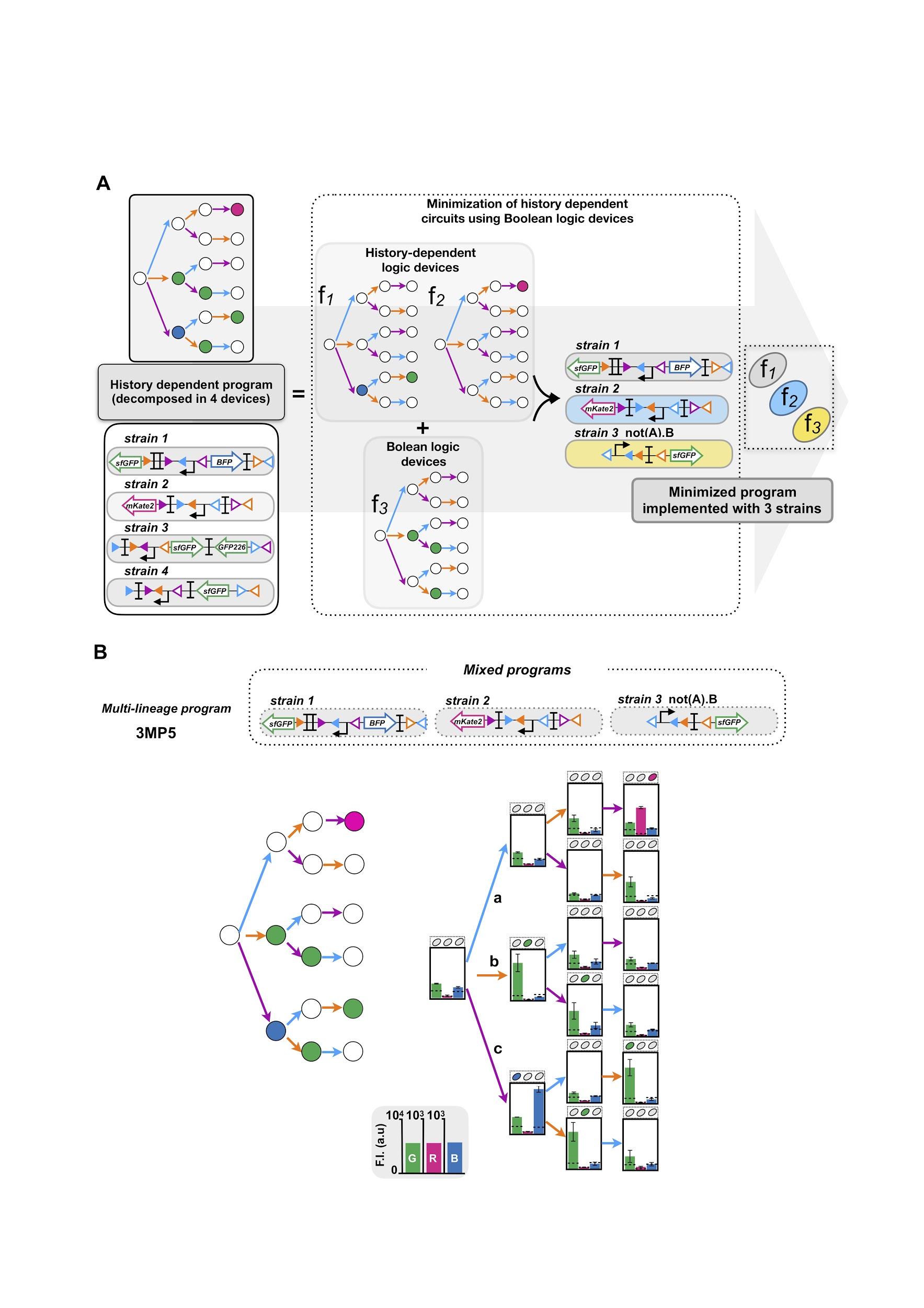
**

**Figure S17. Minimization by simplification of history-dependent programs into Boolean-logic programs.** **(A)** Example of a 3-input program (initially decomposed in 4 sub-programs) that can be simplified into 3 sub-programs using Boolean logic devices. Two subprograms correspond to a lineage tree with 2 and 1 ON states in a single lineage. Each sub-program is implemented in differentes strains. The second subprogram corresponds to a lineage tree with 3 ON states in different lineages simplifiable into a Boolean-logic function (not(A).B), implemented in one strain. Using this minimization scheme, we minimized the required number of strains from 4 to 3. **(B)** Characterization of the 3-input multicellular program simplified on A. The sub-programs were implemented in 3 different strains. The inputs are represented by letters, a for Integrase Bxb1, b for integrase Tp901-1 and c for Int5. The strains were mixed in similar proportions, grown consecutively during 16 hours and sequentially induced with different order-of-occurrences of inputs. Each graph corresponds to a different input state of the lineage tree. The bar graph corresponds to the mean value of fluorescence intensity (F.I) in arbitrary units (a.u) for each fluorescent channel (GFP, RFP and BFP). The error bars correspond to the standard deviation for three biological replicates. The dotted line indicates the negative control autofluorescence.

**Figure S18**

**
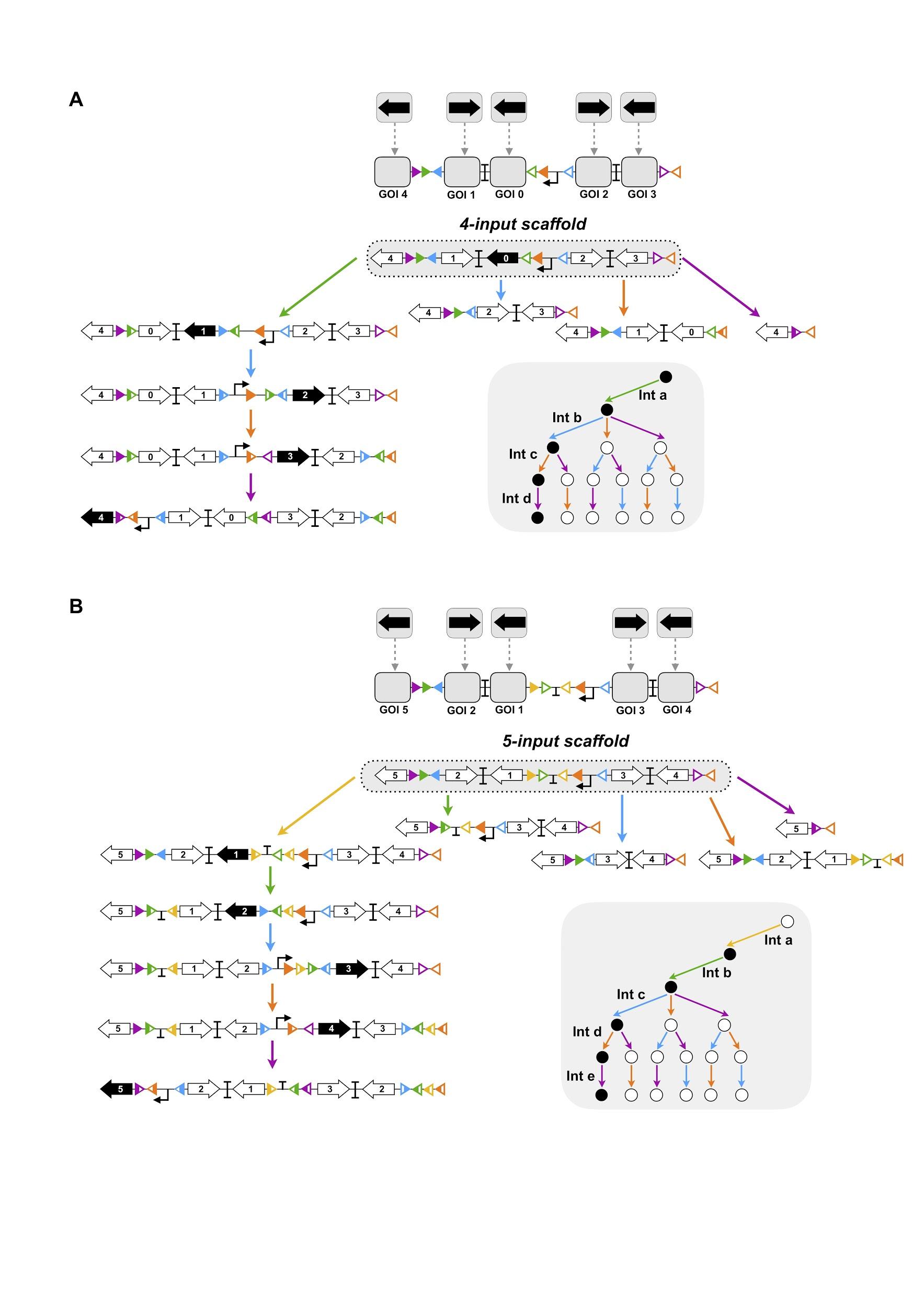
**

**Figure S18. 4 and 5 input scaffold and states diagrams.** 4-input **(A)** and 5-input **(B)** scaffolds. Integrase sites are positioned in each scaffold to permit expression of an output gene in the corresponding lineage. Therefore, for each state of the lineage a different gene is expressed. On the right panel A, gene 0 is expressed only when no input is present. If input a is present first, gene 1 is expressed, but if input b, c or d are present first, none gene is expressed (nor will be expressed) as the promoter is excised. If input b follows input a, gene 2 is expressed and so on. The 5-input scaffold allows expression of a different GOI in each state except in the state 0 (with no input). An additional strain is needed if gene expression is required in this state. Here only intermediate states for the scaffold lineage are represented. For other lineages only the first intermediate state is represented.

**Figure S19**

**
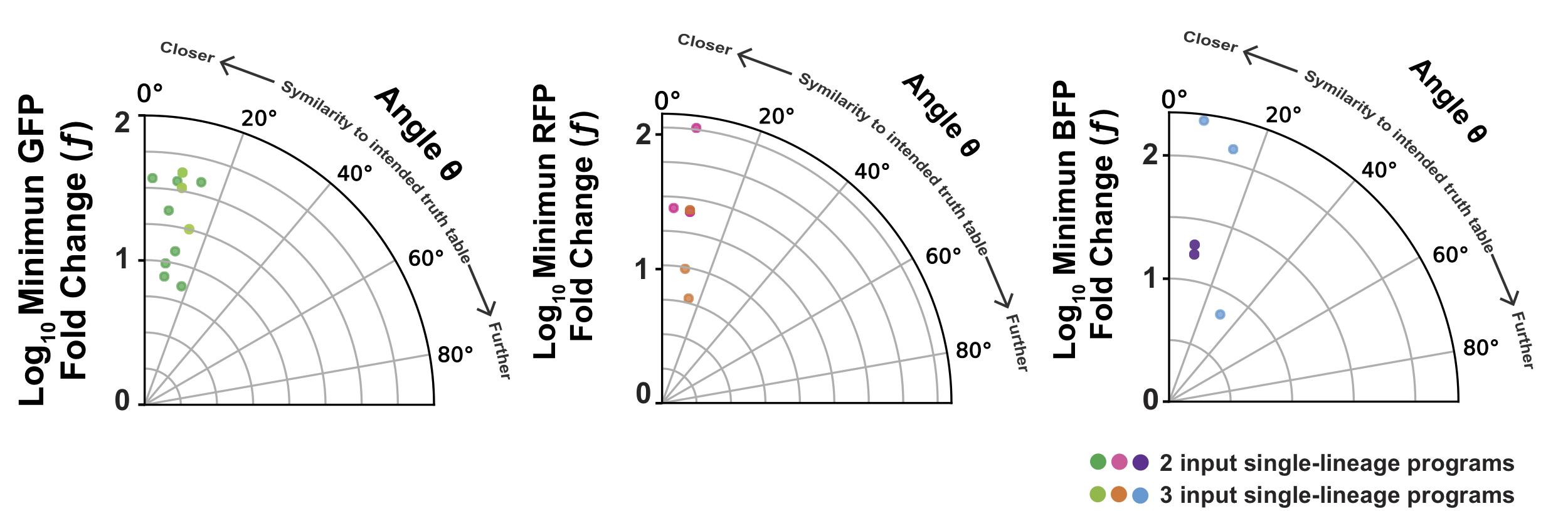
**

**Figure S19. Robustness of history-dependent programs.** Reporter gene expression robustness with angles from fluorescence intensity versus the logarithm of minimum fluorescence fold change (𝒇) for GFP, RFP and BFP, were plotted. The minimum fold change correspond to the fold change between minimum fluorescence in ON input state and the maximum fluorescence in OFF state (𝒇). The *Log_10_* 𝒇 were plotted.

**Figure S20**

**
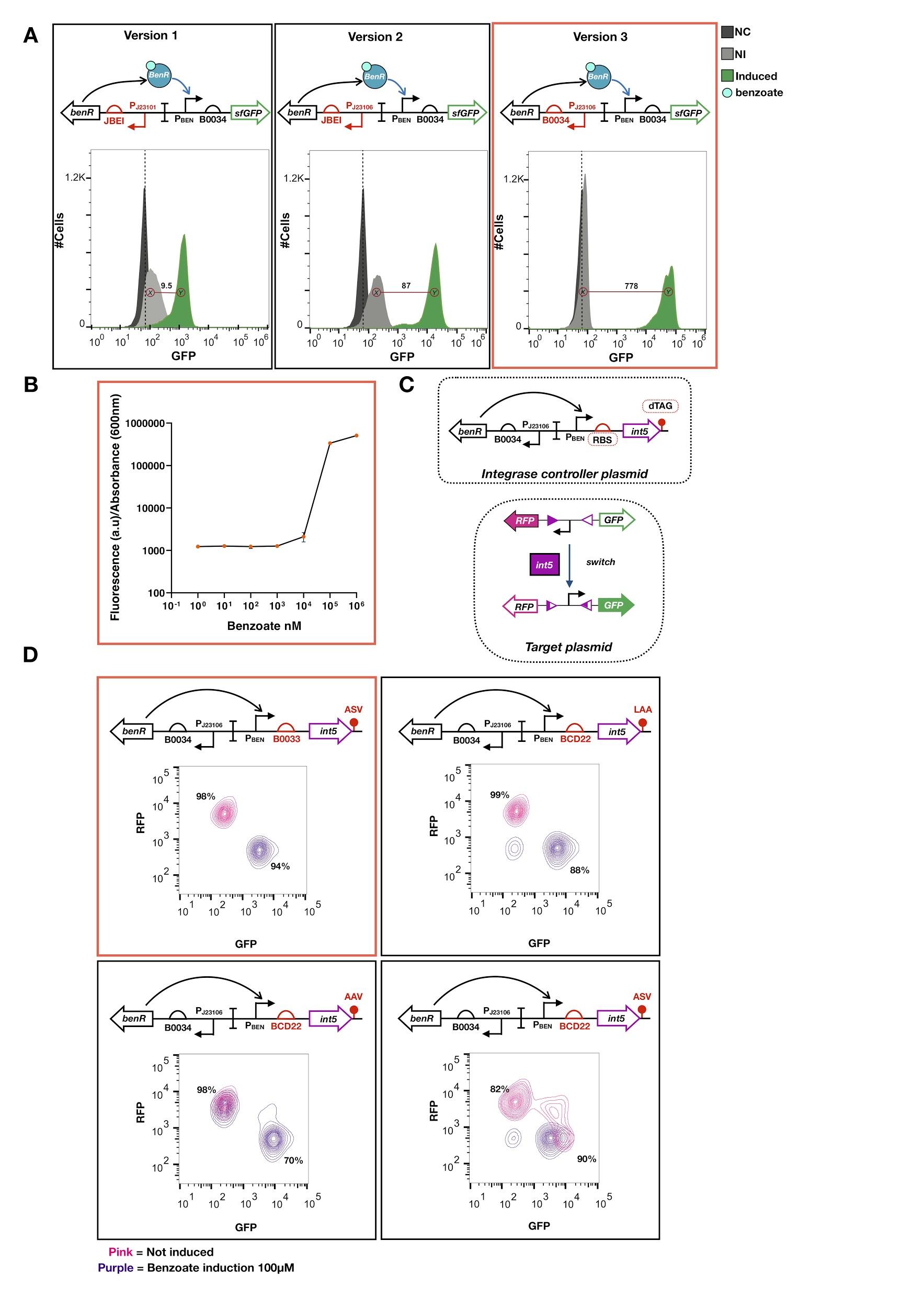
**

**Figure S20. Optimization of BenR and Integrase 5 expression.** Three versions of the benzoate sensor BenR were characterized **(A).** The constructions were designed with different combinations of promoters and RBS to tune *benR* gene transcription, and induce GFP fluorescence in the presence of benzoate. These versions were characterized using 10 μM of benzoate for version 1 and 2 and 100 μM for version 3 as an inductor for 16h at 37°C. Higher concentration affected the growth of cells in versions 1 and 2. The fold change between non-induced cells population (NI, *X* population) and Induced cells population (*Y*) was calculated, for each. Titration curve response of sensor version 3 to benzoate **(B)**. Cells harboring the version 3 of the sensor were induced with different benzoate concentration for 16h. The fluorescence was measured by a plate reader. A maximum fluorescence was observed after 100 μM of benzoate induction. Scheme of Integrase 5 gene expression optimization and target plasmid to test switching efficiency **(C)**. Characterization of different constructions for *int5* gene expression **(D)**. Different RBS and degradation tags were tested. Plots show flow cytometry measurements of fluorescence. Cells not induced (pink) are expressing RFP fluorescence (state 0). After induction with benzoate 100 μM (purple), expression of GFP is observed (state 1). The percentage of cells in each state are shown. Red square indicates the construction selected to use for the integrated triple controller plasmid.

**Figure S21**

**
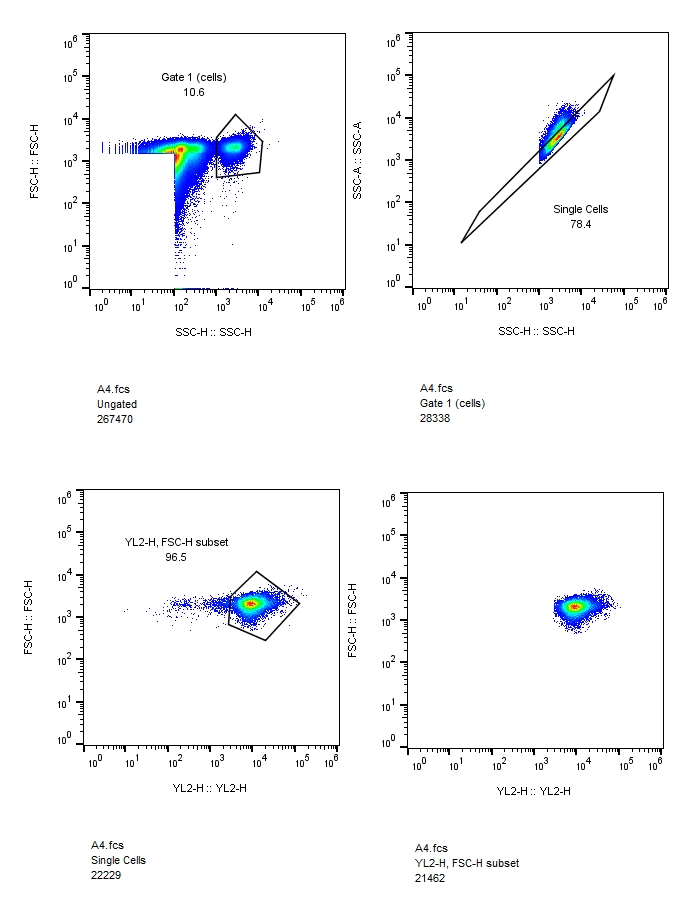
**

**Figure S21. Figure exemplifying the gating strategy.** Gates were designed based on FSC-H vs SSC-H graphs to remove debris from the analysis and SSC-A vs SSC-H to doublet discrimination (lef and right upper panel). Cell subsets expressing the same fluorescent protein were selected with gates based on green, red and blue intensities (bottom panels).

**Figure S22**


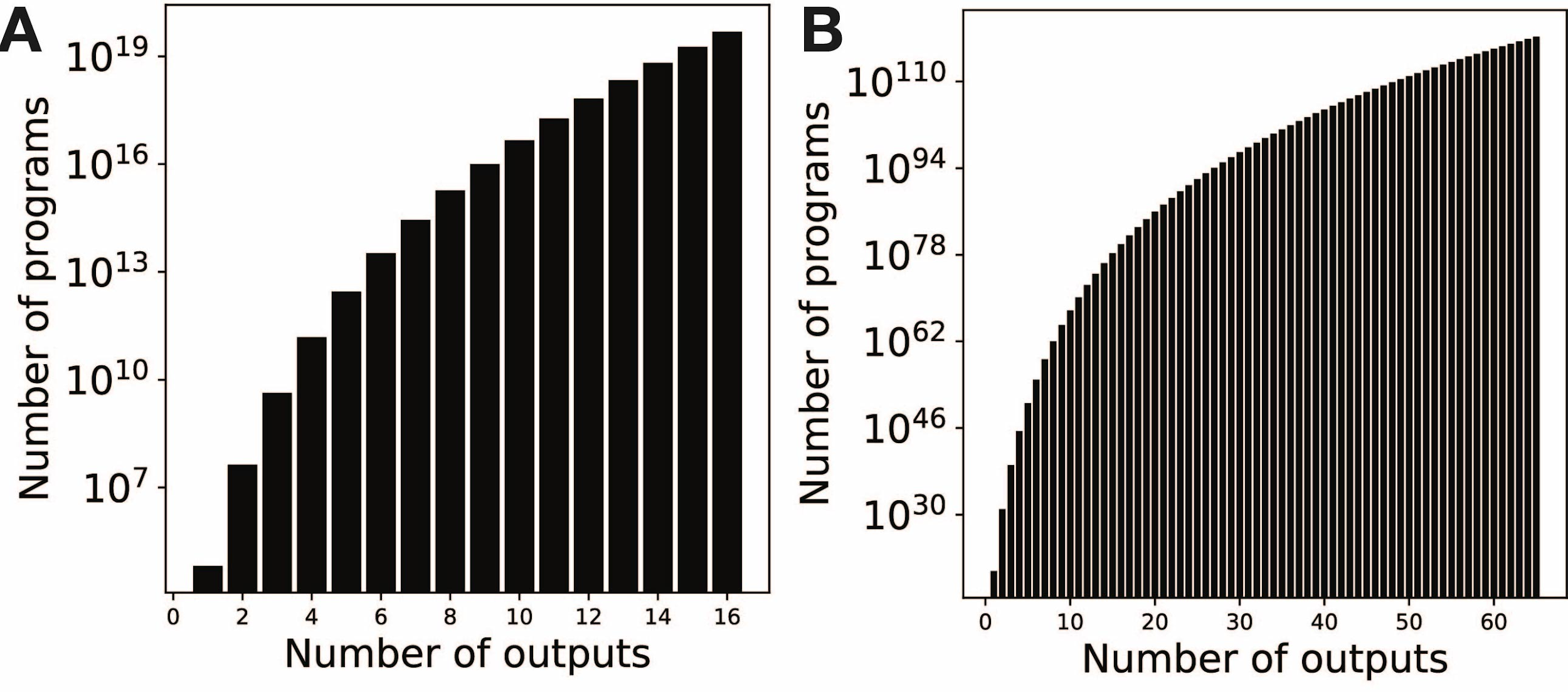


**Figure S22. Number of possible programs for 3- and 4-inputs according to the number of outputs**, with **(A)** for 3-input programs and **(B)** for 4-input programs. The x-axis corresponds to the number of outputs and the y-axis to the number of programs corresponding, and the y-axis is in logarithmic scale.

**Supplementary table 1**

**
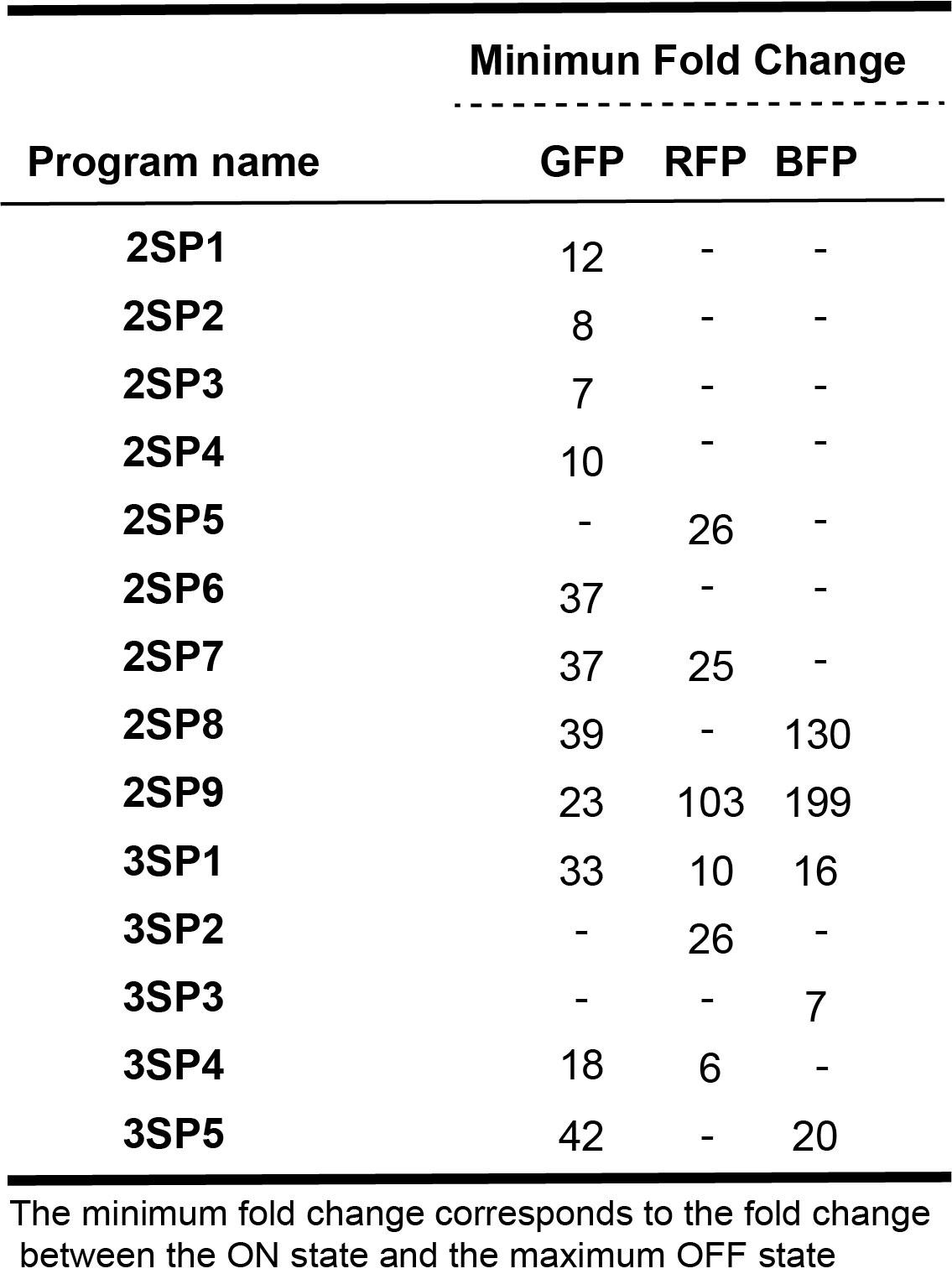
**
