# Supplementary material for: Rational programming of history-dependent logic in cellular populations
Source: Nat Commun. 2020 Sep 21;11:4758. doi: 10.1038/s41467-020-18455-z (PMC7506022; doi:10.1038/s41467-020-18455-z)
Supplement: Supplementary file 3 — Reporting Summary [file 41467_2020_18455_MOESM3_ESM.pdf]

nature research

Corresponding author(s): Jerome Bonnet

Reporting Summary

Nature Research wishes to improve the reproducibility of the work that we publish. This form provides structure for consistency and transparency in reporting. For further information on Nature Research policies, see Authors & Referees and the Editorial Policy Checklist.

Statistical parameters

When statistical analyses are reported, confirm that the following items are present in the relevant location (e.g. figure legend, table legend, main text, or Methods section).

n/a	Confirmed	
	x	The exact sample size ( ) for each experimental group/condition, given as a discrete number and unit of measurement
	x	An indication of whether measurements were taken from distinct samples or whether the same sample was measured repeatedly
x		The statistical test(s) used AND whether they are one- or two-sided<br>*Only common tests should be described solely by name; describe more complex techniques in the Methods section.*
x		A description of all covariates tested
x		A description of any assumptions or corrections, such as tests of normality and adjustment for multiple comparisons
	x	A full description of the statistics including central tendency (e.g. means) or other basic estimates (e.g. regression coefficient) AND variation (e.g. standard deviation) or associated estimates of uncertainty (e.g. confidence intervals)
x		For null hypothesis testing, the test statistic (e.g. *F*, *t*, *r*) with confidence intervals, effect sizes, degrees of freedom and *P* value noted<br>*Give P values as exact values whenever suitable.*
x		For Bayesian analysis, information on the choice of priors and Markov chain Monte Carlo settings
x		For hierarchical and complex designs, identification of the appropriate level for tests and full reporting of outcomes
x		Estimates of effect sizes (e.g. Cohen's *d*, Pearson's *r*), indicating how they were calculated
	x	Clearly defined error bars<br>*State explicitly what error bars represent (e.g. SD, SE, CI)*

Our web collection on statistics for biologists may be useful.

Software and code

Policy information about availability of computer code

Data collection	Flow cytometry data collected using the Attune NxT software version 2.7 and BD LSR Fortessa (Bencton Dickinson) with FACSDiVa v9.0 software.<br>Plate reader data collected the Biotek Gen5 software
Data analysis	Flow cytometry: Flowjo (treestar). Custom python scripts for generating plots available at github (https://github.com/synthetic-biology-group-cbs-montpellier/OSIRiS) CALIN (https://github.com/synthetic-biology-group-cbs-montpellier/calin)<br>Plate reader : microsoft excel. (Fluorescence data was normalized by OD using excel software). Omero software version 5.5.0

For manuscripts utilizing custom algorithms or software that are central to the research but not yet described in published literature, software must be made available to editors/reviewers upon request. We strongly encourage code deposition in a community repository (e.g. GitHub). See the Nature Research guidelines for submitting code & software for further information.

Data

Policy information about availability of data

All manuscripts must include a data availability statement. This statement should provide the following information, where applicable:

- Accession codes, unique identifiers, or web links for publicly available datasets
- A list of figures that have associated raw data
- A description of any restrictions on data availability

Source data for main text figures, along with DNA sequences for all constructs are provided in supplementary materials.

see supplementary excel files and data availabilty section.

Field-specific reporting

Please select the best fit for your research. If you are not sure, read the appropriate sections before making your selection.

- [x] Life sciences
- [ ] Behavioural & social sciences
- [ ] Ecological, evolutionary & environmental sciences

For a reference copy of the document with all sections, see nature.com/authors/policies/ReportingSummary-flat.pdf

Life sciences study design

All studies must disclose on these points even when the disclosure is negative.

Sample size	Each experiment was performed in triplicates. For flow cytometry we analyzed 20000 cells / sample.
Data exclusions	none
Replication	All measurements performed in triplicates three times on three different days. All attempts at replication were successful, and reliable replicated
Randomization	No randomization was done. The randomization was not relevant for our study.
Blinding	No blinding was done. Blinding was not done because it was not conducive to this study

Reporting for specific materials, systems and methods

Materials & experimental systems

n/a	Involved in the study	
x		Unique biological materials
x		Antibodies
x		Eukaryotic cell lines
x		Palaeontology
x		Animals and other organisms
x		Human research participants

Methods

n/a	Involved in the study	
x		ChIP-seq
	x	Flow cytometry
x		MRI-based neuroimaging

Flow Cytometry

Plots

Confirm that:

- [x] The axis labels state the marker and fluorochrome used (e.g. CD4-FITC).
- [x] The axis scales are clearly visible. Include numbers along axes only for bottom left plot of group (a 'group' is an analysis of identical markers).
- [x] All plots are contour plots with outliers or pseudocolor plots.
- [x] A numerical value for number of cells or percentage (with statistics) is provided.

Methodology

Sample preparation	We worked using E. coli cells DH5alphaZ1, which were grown in Azure Hi-Def medium details for analysis are described in materials and methods.
Instrument	Attune Nxt Flow cytometer and BD LSR Fortessa.
Software	For data collection, Attune NxT software version 2.7 and FACSDiVa software. Data analysis FlowJo V10.
Cell population abundance	We analyzed 20000 single cells after having performed gating for removing debis and doublets (see below).
Gating strategy	Quantification of expression levels in all strains was performed using an Attune NxT flow-cytometer (Thermofisher) equipped with an autosampler. Experiments were performed on 96 wells plates with 3 replicates per plates. For flow cytometry measurements, 20 000 bacteria events were analysed. A gate was previously designed based on forward and side scatter graphs to remove debris from the analysis. Doublet discrimination were set with a gate based on SSC-A vs SSC-H. Experiment on Attune NxT were performed in 96 well plates with setting; FSC: 200V, SSC: 380V, green intensity BL1: 460V (488 nm laser and a 510/10 nm filter), red intensity YL2: 460V (561 nm laser and a 615/25 nm filter). Setting for experiments on Fortessa were FSC: 400V, SSC: 300V, green intensity GFP: 580V (488 nm laser and a 530/30 nm filter), red intensity mCherry: 565V (600nm laser and a 610/20 nm filter) and blue intensity V1: 460V (405nm laser and a 450/50 nm filter)<br>Data were analyzed and presented using the Flow-Jo (Tristar) software.

☒ Tick this box to confirm that a figure exemplifying the gating strategy is provided in the Supplementary Information.
