# Supplementary material for: Rational programming of history-dependent logic in cellular populations
Source: Nat Commun. 2020 Sep 21;11:4758. doi: 10.1038/s41467-020-18455-z (PMC7506022; doi:10.1038/s41467-020-18455-z)
Supplement: Supplementary file 5 — Description of Additional Supplementary Files [file 41467_2020_18455_MOESM5_ESM.pdf]

Title: Supplementary Movie 1.

Description: Switching kinetics of 2SP9 Single-lineage program growing in mother machine microfluidic device. Cells were grown in the microfluidic device for 10 h and analyzed by time-lapse microscopy, the merged channels for BFP, RFP and GFP are shown. Cells were grown for 30 min without induction, followed by 3 h of aTc induction. Next, the medium was changed and arabinose induction was performed (induction time at to top of the movie, in hours). (see also figure S6).
